# Flavonoid Phloretin Inhibits Adipogenesis and Increases OPG Expression in Adipocytes Derived from Human Bone-Marrow Mesenchymal Stromal-Cells

**DOI:** 10.3390/nu13114185

**Published:** 2021-11-22

**Authors:** Antonio Casado-Díaz, Ángel Rodríguez-Ramos, Bárbara Torrecillas-Baena, Gabriel Dorado, José Manuel Quesada-Gómez, María Ángeles Gálvez-Moreno

**Affiliations:** 1 Unidad de Gestión Clínica de Endocrinología y Nutrición—GC17, Instituto Maimónides de Investigación Biomédica de Córdoba, Hospital Universitario Reina Sofía, CIBERFES, 14004 Córdoba, Spain; angel.rguezramos@gmail.com (Á.R.-R.); b42tobab@uco.es (B.T.-B.); md1qugoj@uco.es (J.M.Q.-G.); mariaa.galvez.sspa@juntadeandalucia.es (M.Á.G.-M.); 2 Dep. Bioquímica y Biología Molecular, Campus Rabanales C6-1-E17, Campus de Excelencia Internacional Agroalimentario (ceiA3), Universidad de Córdoba, CIBERFES, 14071 Córdoba, Spain; bb1dopeg@uco.es

**Keywords:** phloretin, human mesenchymal stem cells, adipogenesis, obesity, bone

## Abstract

Phloretin (a flavonoid abundant in apple), has antioxidant, anti-inflammatory, and glucose-transporter inhibitory properties. Thus, it has interesting pharmacological and nutraceutical potential. Bone-marrow mesenchymal stem cells (MSC) have high differentiation capacity, being essential for maintaining homeostasis and regenerative capacity in the organism. Yet, they preferentially differentiate into adipocytes instead of osteoblasts with aging. This has a negative impact on bone turnover, remodeling, and formation. We have evaluated the effects of phloretin on human adipogenesis, analyzing MSC induced to differentiate into adipocytes. Expression of adipogenic genes, as well as genes encoding OPG and RANKL (involved in osteoclastogenesis), protein synthesis, lipid-droplets formation, and apoptosis, were studied. Results showed that 10 and 20 µM phloretin inhibited adipogenesis. This effect was mediated by increasing beta-catenin, as well as increasing apoptosis in adipocytes, at late stages of differentiation. In addition, this chemical increased *OPG* gene expression and *OPG*/*RANKL* ratio in adipocytes. These results suggest that this flavonoid (including phloretin-rich foods) has interesting potential for clinical and regenerative-medicine applications. Thus, such chemicals could be used to counteract obesity and prevent bone-marrow adiposity. That is particularly useful to protect bone mass and treat diseases like osteoporosis, which is an epidemic worldwide.

## 1. Introduction

Phloretin (2′,4′,6′-trihydroxy-3-(4-hydroxyphenyl)-propiophenone) is a flavonoid belonging to the chalcone class. It is abundant in fruits such as apple, pear, and strawberries [1,2,3]. Its pharmacological properties include its high antioxidant capacity [4], anti-inflammatory activity [3,5,6], and its ability to inhibit glucose transporters (GLUT) [7]. Thus, phloretin has been proposed for different clinical applications. They include treatment of cardiovascular pathologies, skin diseases, cancer, osteoporosis, diabetes, or obesity [8,9,10,11].

Mesenchymal stem cells (MSC) are progenitor cells with the capacity to differentiate into different cell types. They include osteoblasts, adipocytes, and chondrocytes, among others [12]. MSC play an important role in tissue homeostasis and regeneration [13]. They exert such functions through their capacity to differentiate into other cell types and secrete factors. This way, they can regulate different biological processes such as inflammation, apoptosis, migration, or proliferation [14]. MSC have been localized in different tissues in adults, such as bone marrow, adipose tissue, placental tissue, and hair follicles, among others [15]. Bone marrow and adipose tissue have been the main sources used by researchers for their isolation and study. MSC have interesting immunomodulatory and regenerative properties. Therefore, such cells and their secretome have promising potential for applications in regenerative medicine [14,16]. At the research level, MSC are also widely used as an in vitro model for the study of cell differentiation. Thus, numerous studies with MSC have evaluated how different substances or drugs can affect processes such as osteoblastogenesis, adipogenesis, or chondrogenesis, among others [17,18,19].

MSC are progenitor cells of osteoblasts. With aging, changes in cellular niches of bone marrow induce MSC to differentiate into adipocytes, rather than osteoblasts. This favors an increase of adiposity in bone marrow, bone-mass loss, and increased risk of fractures [20]. In the case of individuals with pathologies such as obesity and type 2 diabetes, increasing adiposity tissue in bone marrow has been related to diseases like osteoporosis [21]. In addition, adipocytes in bone marrow are an important source of nuclear factor -kappa B ligand (RANKL), which is an activator of osteoclast and bone resorption [22]. Thus, an increase in bone-marrow adiposity decreases bone formation capacity, which increases bone-fracture risk. That is due to a decrease in osteoblasts and an increase in bone resorption, due to activation of osteoclastogenesis.

Excessive adipose tissue, mainly white adipose tissue, can lead to obesity. This is characterized by an imbalance between consumed and expended energy [23]. Obesity is currently an epidemic in developed countries. Thus, the World Health Organization estimates that in 2016 there were 1.9 million adults with overweight and 650 million with obesity. Moreover, obesity is a major risk factor for multiple pathologies, including cardiovascular diseases. For this reason, obesity and its derived problems represent an important health expenditure, which has an increasing trend in such countries. Thus, in the United States, spending on obesity-related treatments increased from 6.13% in 2001 to 7.91% in 2015, an increment of 29% [24]. Adipose tissue can spread by hypertrophy, in expanding the size of existing adipocytes, and/or hyperplasia, increasing the number of adipocytes through adipogenesis. In this scenario, different therapies, based on controlling and inhibition of adipogenesis, have been proposed. They take into account the importance of adipogenesis in developing obesity [25]. Among the suggested therapies is the use of natural products derived from plants, particularly those with antioxidant properties [26]. Consumption of phytochemicals or drugs that inhibit differentiation of MSC into adipocytes may have positive effects on the treatment of pathologies such as obesity [27]. Currently, existing pharmaceutical drugs to combat obesity are based on products that act at the neuronal level on appetite, or on hormones such as incretins [28]. Other alternative therapies for the treatment of obesity have also been proposed. They include induction of adipocyte apoptosis, through natural or chemically synthesized compounds [29]. In addition, these treatments could potentially decrease bone-marrow adiposity during aging, having a positive impact on bone metabolism.

Animal studies have shown that phloretin has positive effects on the treatment of obesity [30]. However, there are contradictory results in relation to adipogenesis. Thus, while some reports indicate that phloretin promotes adipogenesis [31,32,33,34], others indicate the opposite [35,36,37]. These studies have been conducted mainly in mouse cell models such as 3T3-L1 and ST2 preadipocytes, and porcine primary adipocytes. However, little is known about the effect of phloretin on the differentiation of MSC of human origin. Thus, the aim of this study was to evaluate the possible effect of such compounds on the in vitro differentiation of human bone marrow-derived MSC into adipocytes.

## 2. Materials and Methods

### 2.1. MSC Culture and Adipogenic Differentiation

Human MSC from bone marrow were obtained from cryopreserved and previously characterized cultures, belonging to our cell collection [38]. MSC were thawed and grown in Alpha Minimum Essential-Medium (α-MEM) from Cambrex Bio Science—Lonza (Basel, Switzerland), containing 2 mM UltraGlutamine (Lonza), 10% fetal bovine serum (FBS) (Gibco—Thermo Fisher Scientific, Waltham, MA, USA), 100 U ampicillin, 0.1 mg streptomycin/mL and 1 ng basic fibroblast-growth factor (bFGF)/mL from Sigma-Aldrich (Saint Louis, MO, USA). This medium has a glucose concentration of 5.5 mM. In some experiments and treatments, the culture medium was supplemented with glucose up to a concentration of 25 mM. Cells were seeded in 75 cm^2^ flasks from Nalgene-Nunc—Thermo Fisher Scientific and incubated at 37 °C with 5% CO_2_ and 95% humidity. Culture media were changed every 3 to 4 days.

When the culture reached near 90% of confluence, cells were detached with trypsin-EDTA (Gibco) and seeded in culture plates (Nalgene-Nunc-Thermo Fisher Scientific) at a density of about 1000 cells/cm^2^. Once confluence between 70 and 90% was reached, the MSC were induced to differentiate into adipocytes, using the following adipogenic medium: culture medium without bFGF supplemented with 5 × 10^−7^ M dexamethasone, 50 μM indomethacin, and 0.5 mM isobutylmethylxanthine. Experimental designs included treatment of MSC induced or not induced to differentiate into adipocytes, with different concentrations of phloretin (Sigma-Aldrich, Saint Louis, MO, USA). Control cells were treated with a vehicle (ethanol) instead of a flavonoid.

### 2.2. Cell Viability

Cell viability was determined using 3-(4, 5-dimethylthiazolyl-2)-2,5-diphenyltetrazolium bromide (MTT) (Sigma-Aldrich). MSC were seeded at a density of 4000 cells per well, in 96-well plates, and incubated for 24 h. Subsequently, MSC were treated with different concentrations of phloretin. At 48 h, the medium was removed and 100 µL of MEMα medium supplemented with 1 mg MTT/mL was added. After 2 h of incubation in culture conditions, the solution was removed. Insoluble formazan crystals produced were dissolved in isopropanol. In the resulting solution, absorbance at 570 nm was measured, using absorbance at 650 nm as a reference, with a PowerWave XS microplate spectrophotometer from BioTek Instruments (Winooski, VT, USA). In some experiments, undifferentiated cells, or cells differentiated to adipocytes, were maintained for 6 or 13 days. Then, the culture medium was replaced with a medium plus MTT, for viability quantification, as described above.

### 2.3. Glucose Uptake

To evaluate glucose uptake in adipocyte cultures, treated or not with different concentrations of phloretin, the culture medium was removed. Then, wells were washed twice with phosphate-buffered saline (PBS). Cells were subsequently maintained under culture conditions, in PBS + 0.5% bovine serum albumin (BSA), for 5 h. After that, medium was removed and PBS + 0.5% BSA supplemented with 80 µM of 2-deoxy-2-[(7-nitro-2,1,3-benzoxadiazol-4-yl)amino]-D-glucose (2-NBDG) (Sigma-Aldrich) was added. After one hour, cells were washed twice with PBS. Fluorescence was quantified at 485 nm excitation and 535 emissions, using an Infinite F200 Pro fluorometer from Tecan (Mannedorf, Switzerland).

### 2.4. Oil Red-O Staining

The formation of lipid droplets in cultures induced to differentiate into adipocytes was evaluated by oil red-O staining. Cultures were fixed with 3.7% formaldehyde for 15 min. Then they were stained with a solution of 60% of oil red-O (using a stock solution of oil-red-O at 0.35% (*w/v*, in isopropanol)) in distilled water. After 20 min of incubation, cells were washed with distilled water and stained with hematoxylin. From each well of P24 plates, at least nine optical microscopic images at 200× were randomly taken. They were analyzed by ImageJ software (version 1.53f51) from the National Institutes of Health (NIH; Bethesda, MD, USA) <https://imagej.nih.gov/ij>, for staining quantification. In each image, stained areas were normalized with the number of cells (oil red-O area/cell number).

### 2.5. Quantification of Gene Expression by Quantitative Real-Time PCR

RNA was isolated using NZY total RNA Isolation Kit from NZYTech (Lisbon, Portugal), following the manufacturer’s instructions. Nucleic acids were quantified with a NanoDrop ND-1000 Spectrophotometer from Thermo Fisher Scientific. Next, up to 900 ng of RNA were retrotranscribed into cDNA, using an iScript cDNA Synthesis Kit from Bio-Rad (Hercules, CA, USA), according to the manufacturer’s directions.

Quantitative real-time PCR (QRT-PCR) was carried out on a LightCycler 96 Instrument from Roche Applied Science (Penzberg, Germany). Each PCR reaction was performed in a 10 µL volume containing 1 µL of cDNA, 10 pmoles of each primer pair (Table 1), and 1X of SensiFAST Sybr No-Rox Mix from Bioline (London, UK). The PCR amplification program included one cycle at 95 °C for 2 min (DNA denaturation) and 40 to 45 cycles of 95 °C for 5 s (DNA denaturation) and 65 °C for 30 s (primer hybridization and extension by DNA polymerase). Results were analyzed with LightCycler 1.1 software from the same manufacturer. RNA polymerase II (targeted DNA) polypeptide A (POLR2A) was used as a constitutive housekeeping gene.

### 2.6. Western Blot

Cells were lysed with Cell Extraction Buffer (Thermo Fisher Scientific, Waltham, MA, USA) supplemented with 1 mM of phenylmethylsulfonyl fluoride (PMSF) and 50 μL/mL of protease inhibitor cocktail (PIC) (both from Sigma-Aldrich). The collected lysate was incubated in ice for 30 min, with vortex agitation every 10 min. Finally, it was centrifuged for 10 min (13,000 g) at 4 °C. Cellular-debris precipitate was discarded. The supernatant was transferred into a new tube and stored at −20 °C until used. Protein concentration was quantified with the Bio-Rad DC Protein Assay Kit (Bio-Rad), according to the manufacturer’s instructions.

Subsequently, 15–20 μg of protein from each sample was loaded into an 8–16% acrylamide nUView Tris-Glycine Precast Gel from NuSeP (Germantown, MD, USA) under denaturing conditions. Electrophoresis was carried out in a Mini-Protean (Bio-Rad) system. Then, the proteins were transferred into a polyvinylidene difluoride (PVDF) membrane (Bio-Rad), using a Trans-Blot Turbo Transfer System from the same manufacturer. Then, membranes were blocked with a 5% solution of skimmed milk, in Tris-Tween Buffered Saline (TTBS) buffer (20 mM Tris-HCl pH 7.6, 150 mM NaCl, and 0.05% Tween) for 1 h at room temperature. Subsequently, membranes were incubated overnight at 4 °C, using primary antibodies anti-beta-catenin (1:1000) and anti-cyclin D1 (1:1500), both from Cell Signaling Technology (Danvers, MA, USA), as well as anti-alpha-tubulin (1:1000) from Abcam (Cambridge, UK), or anti-GAPDH (1:1000), from Santa Cruz Biotechnology (Dallas, TX, USA), in 1% milk in TTBS. After incubation, the antibodies were removed, the membranes were washed with TTBS and incubated with the secondary anti-Rabbit IgG heavy and light (H&L) chains–horseradish peroxidase (HRP) antibody (1:3000), for beta-catenin and cyclin D1, from Abcam (Cambridge, UK) in 1% milk in TTBS for 1 h. A secondary anti-mouse IgG HRP (1:2000) from Santa Cruz Biotechnology was used to detect alpha-tubulin and GAPDH. Finally, membranes were washed with TTBS buffer. They were revealed with Clarity Western ECL Substrate (Bio-Rad) and visualized in a ChemiDoc XRS+ Gel Imaging System from Bio-Rad. Acquisition and analyses of images were performed using Image Lab software version 6.0 from the same company.

Gels used for Western blotting allow the use of stain-free technology. Through the activation of gels with UV light in the ChemiDoc XRS+ Gel Imaging System, images of total protein loaded for each sample were obtained, being quantified with Image Lab software. Values obtained were used for normalization of band intensity, corresponding to studied proteins [39,40].

### 2.7. Detection of Apoptotic Cells by Means of Caspase 3/7-Positives

CellEvent Caspase-3/7 green detection reagent for apoptosis (Life Technologies—Thermo Fisher Scientific, Waltham, MA, USA) was used to detect apoptotic cells. The culture medium was removed and 4 µM of CellEvent Caspase-3/7 green detection reagent in PBS + 5% FBS was added. After 1 h, cells were fixed with 3.7% formaldehyde, permeabilized with PBS + 0.5% triton X-100, and nuclei stained with Hoechst. Images were taken with a Nikon Eclipse Ti fluorescence microscope and analyzed with Image J software. Caspase-3/7-positive cells were normalized in each image with the total number of nuclei.

### 2.8. Statistical Analyses

GraphPad Prism 8.0 program from GraphPad Software (San Diego, CA, USA) was used for statistical analyses. Comparison of data from different treatments was performed using analysis of variance (ANOVA) test, to detect significant changes. That was followed by a Dunnett test to identify significant differences between control and treatments, or Tukey’s test to identify significant differences between pairs of treatments. Differences were considered statistically significant when *p* < 0.05. At least three replicates per parameter studied were analyzed. All graphs show the mean plus standard error of the mean (mean ± SEM).

## 3. Results

### 3.1. Effect of Phloretin on MSC Viability

**Figure 1:**
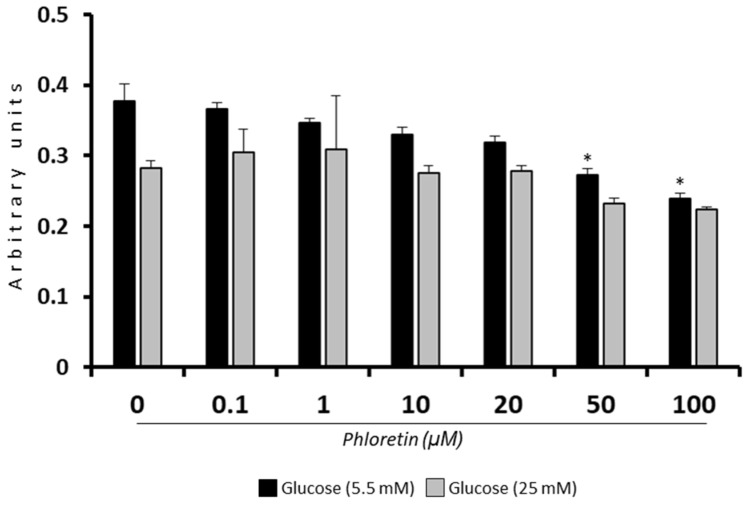
Effect of phloretin concentration on MSC viability in presence of 5.5 or 25 mM of glucose. * *p* < 0.05 vs. untreated cultures (0 µM of phloretin).

In order to determine whether the concentration of phloretin in culture media could affect viability, MSC cultures were treated with concentrations ranging from 0 to 100 µM phloretin for 48 h. Because such chemicals can decrease glucose uptake into cells, and thus affect their metabolism and viability, an assay was performed under normal glucose (NG; 5.5 mM) and high glucose (HG; 25 mM) conditions. Under NG conditions, there was a decrease in viability at concentrations greater than or equal to 50 µM. However, in HG cultures, although a decrease in viability was observed with phloretin concentration, it was not statistically significant with any of the concentrations used (Figure 1). These results indicate that the presence of HG partially protects cells from high concentrations of phloretin in the medium. Therefore, concentrations of 1, 10, and 20 µM phloretin were selected for further experiments.

### 3.2. Phloretin Inhibits MSC Adipogenic Differentiation

**Figure 2:**
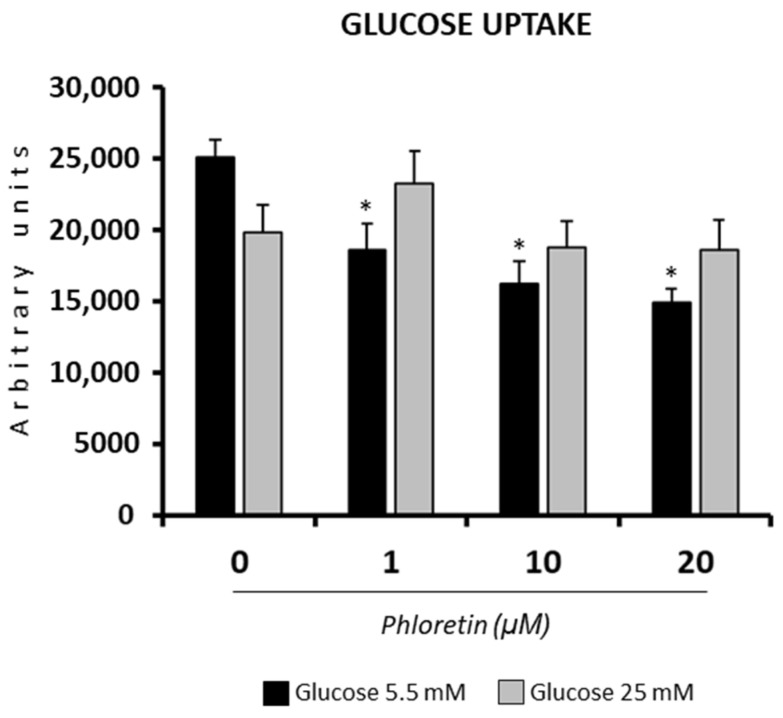
Effect of phloretin on glucose uptake, in MSC differentiated into adipocytes, at day 13 after differentiation started. The assay was performed on cells grown in a medium containing 5.5 or 25 mM of glucose. * *p* < 0.05 vs. cultures no treated (0 µM of phloretin).

Under NG conditions, as expected, the presence of phloretin significantly decreased glucose uptake into adipocyte-induced MSC at day 13. In HG, although a trend toward lower glucose uptake was observed in cells treated with the highest concentrations of phloretin, the changes were not statistically significant (Figure 2).

**Figure 3:**
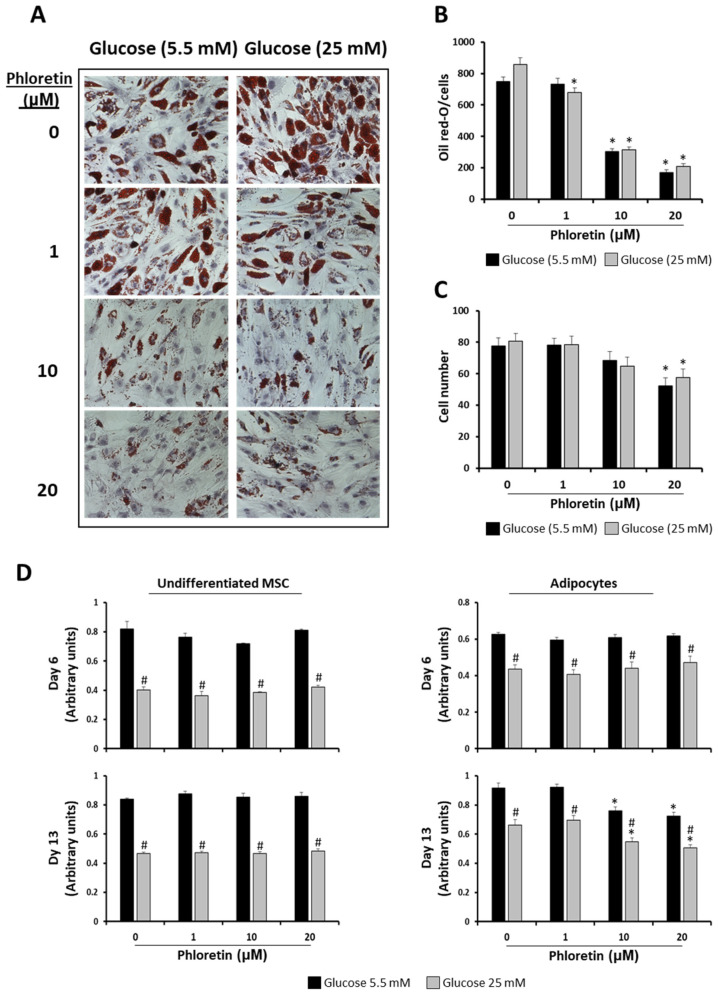
Phloretin decreases lipid accumulation and viability of MSC differentiated into adipocytes. (**A**) Representative light-microscopy pictures (200X) of oil red-O staining of lipid droplets, at day 13 of adipocyte differentiation, in presence of different concentrations of phloretin and glucose. (**B**) Results of quantification of oil red-O by image analyses. (**C**) Cell-number estimation in cultures by image analyses. (**D**) Viability at days 6 and 13 of undifferentiated and differentiating MSC into adipocytes, treated with different phloretin concentrations in medium containing 5.5 or 25 mM of glucose. * *p* < 0.05 vs. cultures not treated (0 µM of phloretin). # *p* < 0.05 vs. cultures in 5.5 mM of glucose treated with the same phloretin concentration.

However, in both NG and HG conditions, the two highest concentrations of phloretin used (10 and 20 µM) significantly inhibited lipid-droplets formation (Figure 3A,B). For quantification of oil red-O staining, images were randomly taken from culture plates and analyzed, as described in materials and methods. For normalization of the quantification of oil red-O staining in each of the images, the number of cells was scored and analyzed. Results showed that the presence of phloretin in the adipogenic medium was associated with a lower number of cells per field analyzed, mainly with the higher concentrations. This decrease was significant with 20 µM phloretin, both in NG and HG, with no statistically significant differences between both conditions of glucose concentrations (Figure 3C).

Considering the above data and in order to test whether phloretin affected MSC viability during adipogenic differentiation, viability was studied at days 6 and 13 after the start of adipogenic differentiation, including non-induced controls. In the latter, tested concentrations of phloretin did not affect viability at either of the two times studied. Nevertheless, MSC maintained in HG medium had in all cases lower viability than those maintained in NG (Figure 3D). In MSC induced into adipocytes, the viability in all treatments and times was also lower in cells maintained in HG compared to those in NG. In both differentiated and undifferentiated MSC, phloretin treatments did not affect viability at day 6. However, at day 13, the two highest concentrations of phloretin tested (10 and 20 µM) significantly decreased the viability of cultures differentiated into adipocytes in both NG and HG (Figure 3D). This positively correlates with the observed decreased cell number, as shown in Figure 3C.

**Figure 4:**
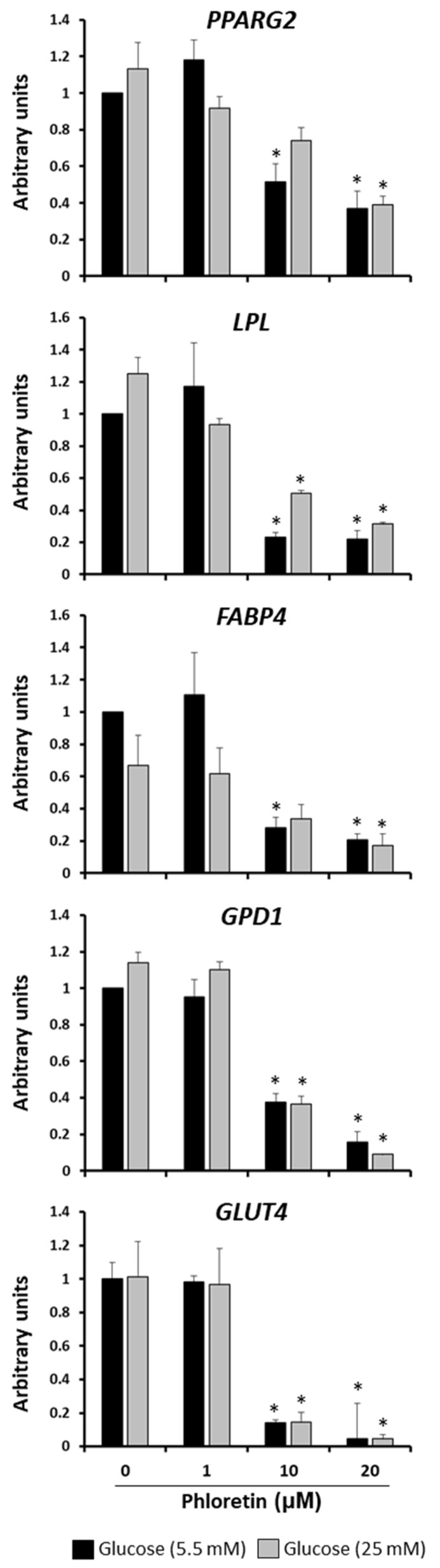
Gene expression of adipogenic marker genes, 13 days after beginning adipogenic differentiation of MSC cultures, in presence of 0, 1, 10, and 20 µM phloretin and 5.5 or 25 mM glucose. * *p* < 0.05 vs. cultures no treated (0 µM of phloretin).

Gene expression of adipogenic genes in MSC cultures induced to adipocytes in the presence or absence of phloretin was also evaluated at day 13. The genes evaluated were peroxisome proliferator-activated receptor gamma 2 (*PPARG2*), lipoprotein lipase (*LPL*), fatty-acid-binding protein 4 (*FABP4*), glycerol-3-phosphate dehydrogenase 1(*GPD1*), and solute carrier family 2 member 4 (*GLUT4*). PPARG2 is a master transcription factor in adipocyte differentiation; LPL, FABP4, and GPD1 are involved in lipid metabolism and GLUT4 encodes an insulin-regulated transporter of glucose. All of them were downregulated in the presence of 10 and 20 µM phloretin in the two glucose concentrations tested (Figure 4). This is consistent with the decrease in lipid droplets observed with the same treatments, as shown in Figure 3. Because the effect of phloretin on adipogenesis was the same in NG as in HG, only NG-grown cultures were used for subsequent experiments.

### 3.3. Phloretin Induce A-Tubulin and Β-Catenin Expression in MSC Differentiated into Adipocytes

**Figure 5:**
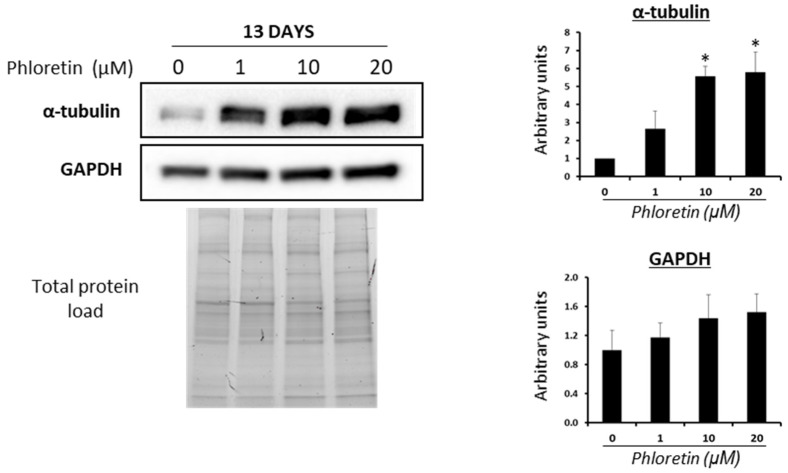
Western blot for α-tubulin and GAPDH of cultures treated with different concentrations of phloretin, at day 13 of adipogenic differentiation. Results of quantification of Western blot bands are shown in the graphs. Protein loading was used for the normalization of values. * *p* < 0.05 vs. cultures no treated (0 µM of phloretin).

Modulation of cytoskeleton and the Wnt/beta-catenin pathway plays a very important role in MSC differentiation into adipocytes. Cell cytoskeleton undergoes remodeling during adipogenesis, allowing accumulation of lipid droplets in the cytoplasm. Among the proteins that are regulated in this process are tubulins [41]. Analyses of α-tubulin protein expression in MSC cultures differentiated into adipocytes, in the presence of phloretin, showed a significant increase in expression with 10 and 20 µM treatments at day 13 (Figure 5). This is another fact indicative of the inhibitory effect of phloretin on adipogenesis. In these experiments, the expression of glyceraldehyde-3-phosphate dehydrogenase (GAPDH), as a possible housekeeping protein, was also studied [42]. As can be seen in Figure 5, quantification of GAPDH expression shows a tendency to increase with the concentration of phloretin in culture medium, albeit not being statistically significant.

**Figure 6:**
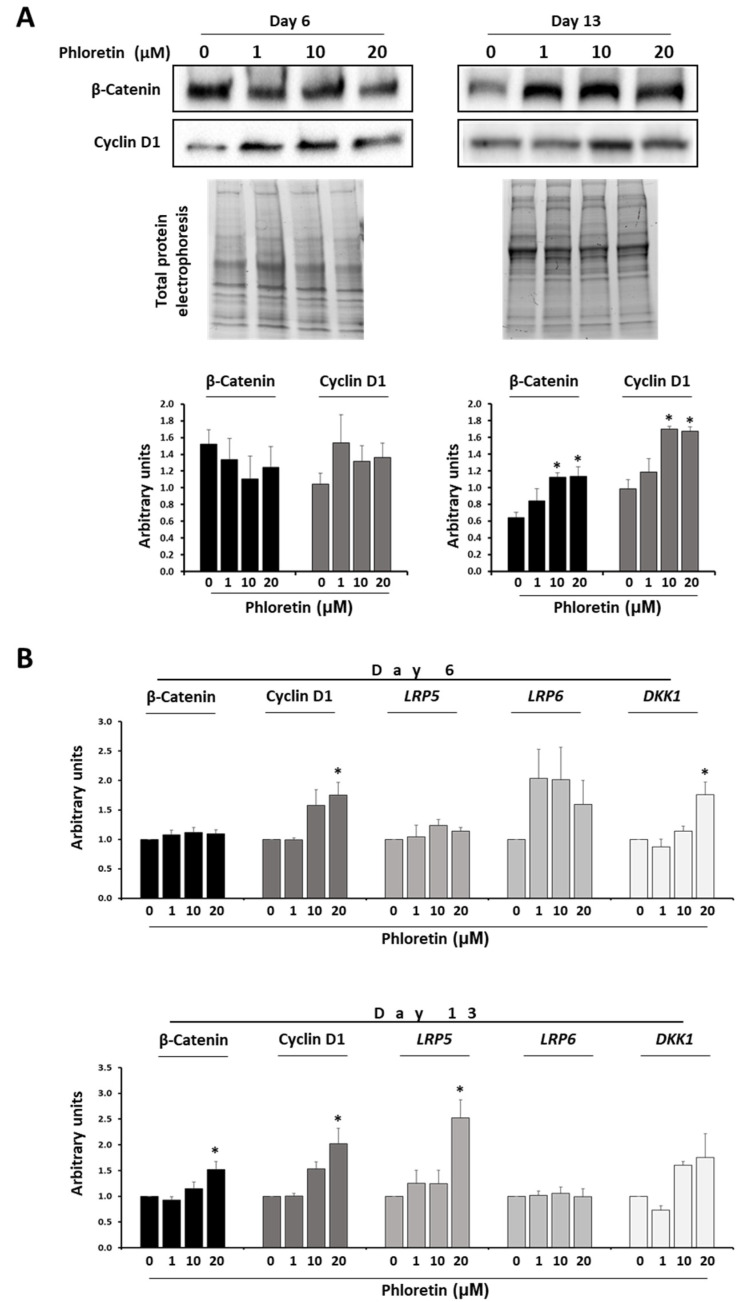
Phloretin affects the Wnt/β-catenin pathway. (**A**) Protein levels of β-catenin and cyclin D1 by Western-blot, at days 6 and 13 of adipogenic differentiation, in presence of 0, 1, 10, and 20 µM of phloretin. Results of the quantification of Western blot bands are shown in the graphs. (**B**) Gene expression of β-catenin, cyclin D1, LRP5, LRP6, and DKK1 (related to the Wnt/β-catenin pathway), at days 6 and 13 of culture treatment. * *p* < 0.05 vs. cultures no treated (0 µM of phloretin).

Regarding the role of the Wnt/beta-catenin pathway in MSC, its activation generally induces osteoblastic gene expression, while repressing differentiation into adipocytes [43]. Because phloretin inhibited adipogenesis, we evaluated whether this could be mediated by beta-catenin. Protein expression studies showed that at day 6 after the start of adipogenic differentiation, the presence of phloretin in the medium had no significant effect on protein expression. However, at day 13 of differentiation, higher concentrations of phloretin produced an increase in protein levels, mainly at 10 and 20 µM (Figure 6A). At the gene expression level, the results for the beta-catenin coding gene were similar. A significant increase in mRNA levels was observed at day 13 with the highest concentration of phloretin (Figure 6B). On the other hand, cyclin D1 is a downstream target of beta-catenin. In parallel to the induction of beta-catenin expression, increased protein synthesis of cyclin D1 in adipocyte cultures treated with 10 and 20 µM phloretin were observed at day 13 (Figure 6A). The mRNA levels of its coding gene (*CCND1*) increased at days 6 and 13 of adipogenic differentiation, in cultures treated with the highest concentration of phloretin (Figure 6B).

Low-density lipoprotein receptor-related proteins 5 and 6 (LRP5 and LRP6) are key components of the LRP5/LRP6/Frizzled co-receptor group, being involved in the canonical Wnt/ beta-catenin pathway [44]. On the other hand, Dickkopf-related protein 1 (DKK1) is an antagonist of the Wnt/β-catenin pathway that acts by preventing LRP5/LRP6 co-receptors to activate the WNT signaling pathway [44]. Gene expression of genes encoding LRP5, LRP6, and DKK1 during adipogenesis of MSC treated with phloretin tend to increase expression in the presence of the flavonoid at day 6. However, only in the *DKK1* gene was a statistically significant increase observed with the highest concentration of phloretin. However, at day 13, significant changes were only observed for *LRP5*. Such gene was induced with 20 µM phloretin treatment (Figure 6B).

### 3.4. Phloretin Decreases Lipid Accumulation in Mature MSC-Derived Adipocytes

**Figure 7:**
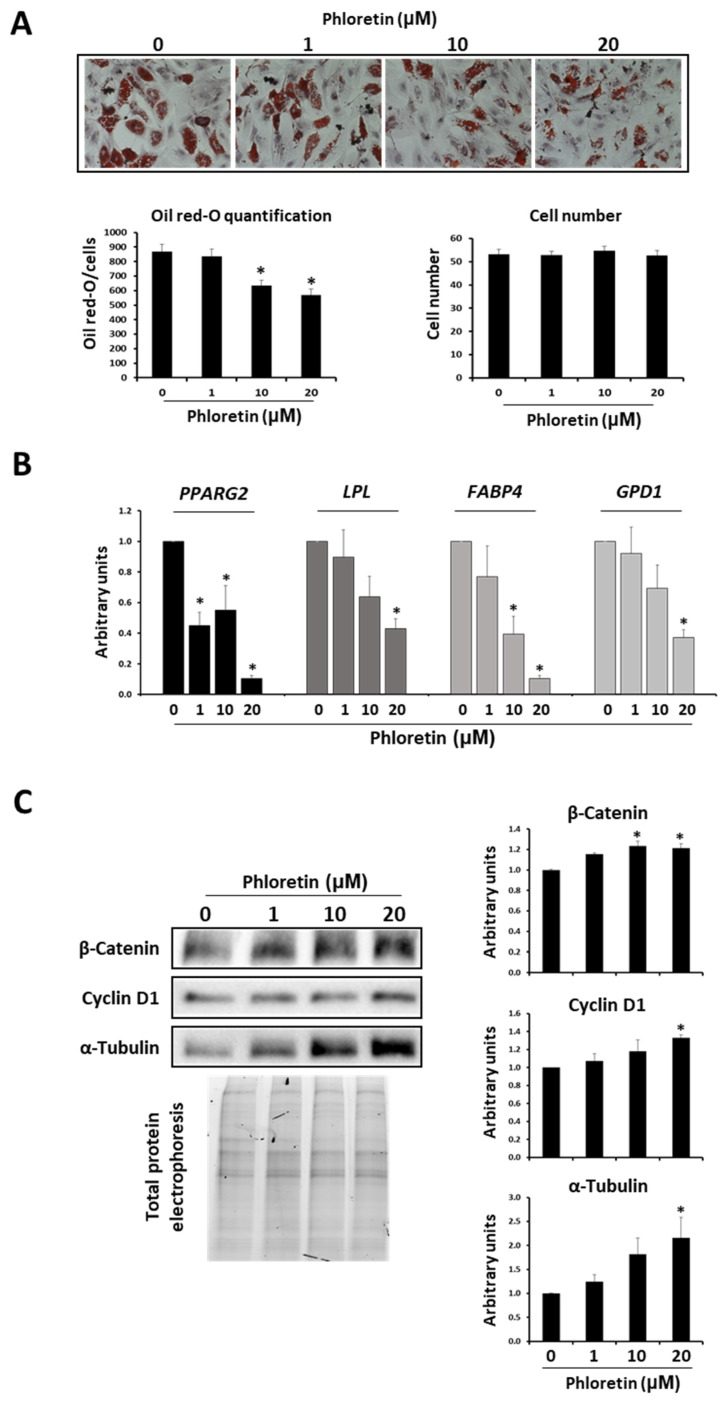
Effects of phloretin on adipocytes treated for 5 days from day 13 of the start of differentiation. (**A**) Representative light-microscopy pictures (200X) of oil red-O staining of cultures treated with different phloretin concentrations. Graphs show oil red-O quantification and cell-number estimation by image analyses. (**B**) Gene expression of adipogenic marker genes (*PPARG2*, *LPL*, *FABP4*, and *GPD1*). (**C**) Western blot for β-catenin, cyclin D1, and α-tubulin, in cultures of adipocytes treated with 0, 1, 10, or 20 µM of phloretin at days 13 to 18 of adipogenic differentiation. * *p* < 0.05 vs. cultures no treated (0 µM of phloretin).

The results described above show that phloretin inhibits adipogenesis when it is present in the culture medium from the beginning of adipogenic differentiation. However, it would also be interesting to evaluate the effect of phloretin on already differentiated adipocytes. For this purpose, MSC were induced to differentiate into adipocytes for 13 days. At that time, cells with a large accumulation of lipid droplets in the cytoplasm were observed, which can be considered mature adipocytes. These cells were then treated with different concentrations of phloretin (0, 1, 10, and 20 µM) for 5 days. Then, lipid-droplet content, adipogenic gene expression, and protein levels of beta-catenin and alpha-tubulin were evaluated. Oil red-O staining showed that lipid accumulation was decreased in cultures treated with 10 and 20 µM phloretin. However, this effect of phloretin treatment was not accompanied by a decrease in the number of cells in the cultures (Figure 7A). In relation to the reduction of lipid content, expression of *PPARG2*, *LPL*, *FABP4*, and *GPD1* adipogenic genes significantly decreased with the higher concentrations of phloretin (Figure 7B). In addition, an increase in beta-catenin and cyclin D1 protein levels was observed in phloretin-treated cultures (Figure 7C). Additionally, along with the decrease in lipid content in adipocytes treated with 10 and 20 µM phloretin, there was an increase of alpha-tubulin, mainly with the higher concentration (Figure 7C).

### 3.5. Phloretin Induces Apoptosis of MSC Differentiated into Adipocytes

**Figure 8:**
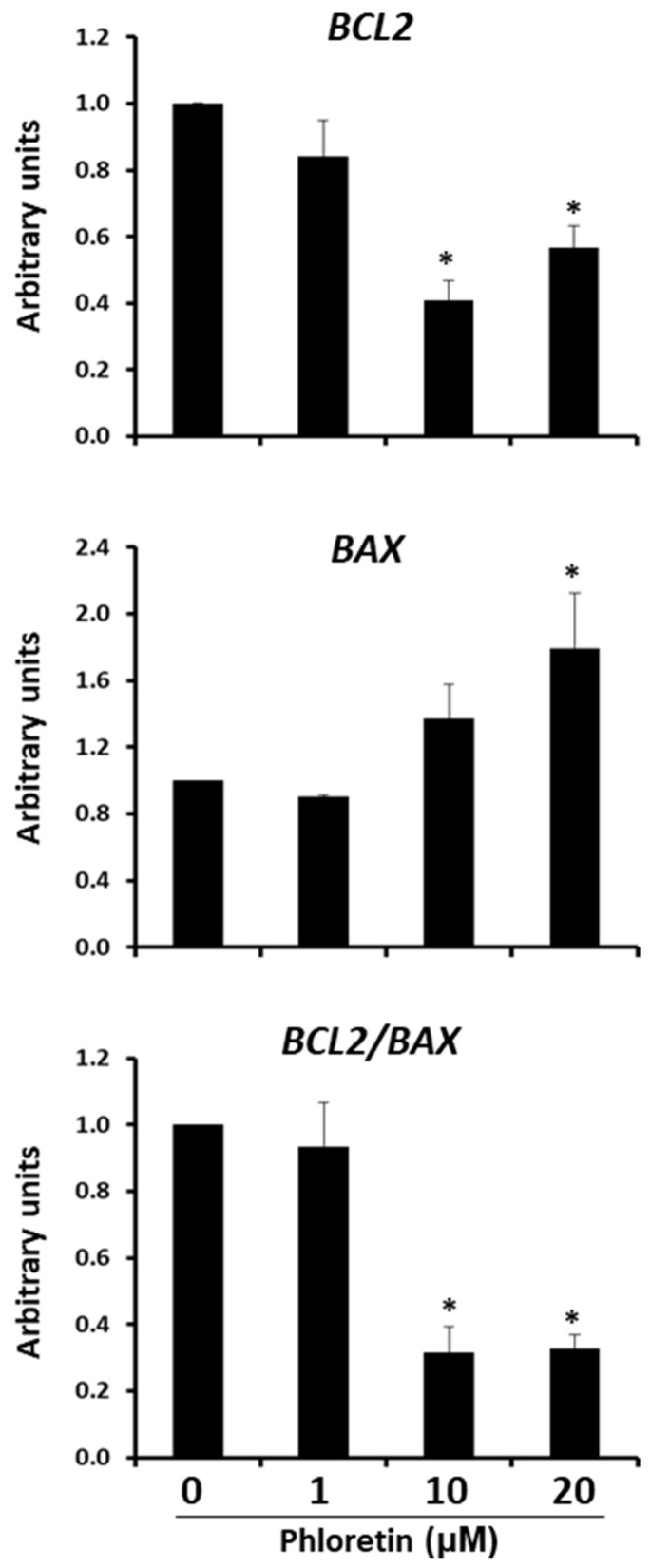
Gene expression of *BCL2* anti-apoptotic and *BAX* apoptotic genes, 13 days after beginning adipogenic differentiation of MSC cultures, in presence of 0, 1, 10, and 20 µM of phloretin. *BCL2/BAX* ratio is also represented. * *p* < 0.05 vs. cultures no treated (0 µM of phloretin).

As previously described, inhibition of adipogenesis by phloretin is accompanied by a decrease in cell viability and cell number in cultures. These effects suggested that phloretin might be inducing apoptosis of these cells. To test this possibility, the expression of *BCL2* anti-apoptotic and *BAX* pro-apoptotic genes was quantified at day 13 after the start of adipogenic differentiation. As shown in Figure 8, expression of *BCL2* gene decreased with the concentration of phloretin in the medium, while that *BAX* was induced. This leads to a significantly lower ratio of *BCL2/BAX* expression in cultures treated with 10 and 20 µM phloretin (Figure 8).

**Figure 9:**
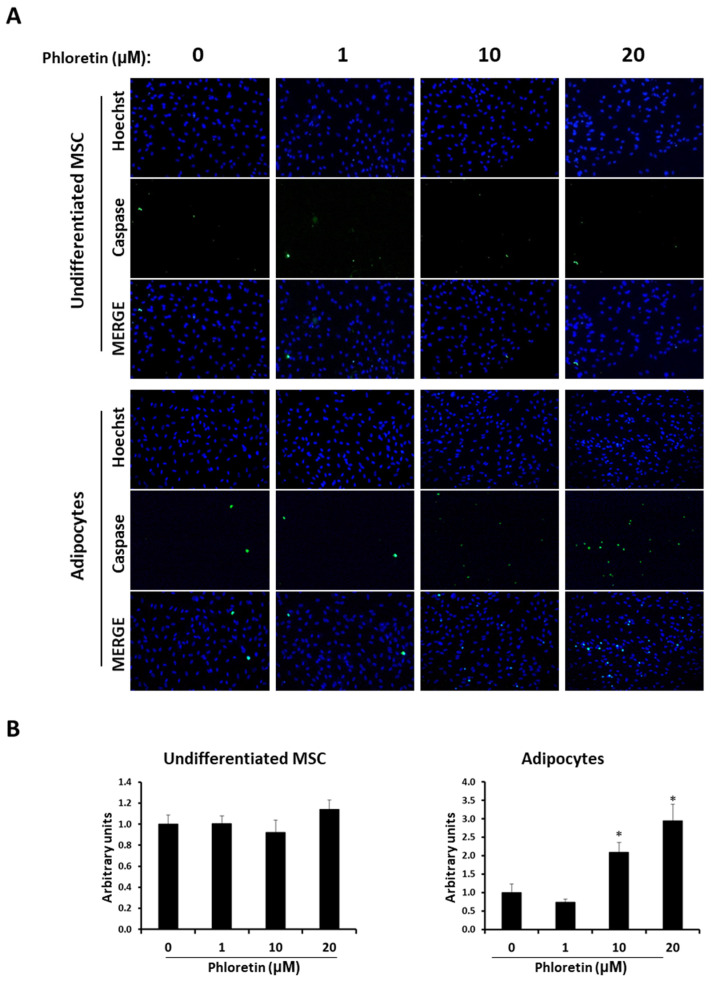
Phloretin increases apoptosis in MSC differentiated into adipocytes. (**A**) Representative fluorescent-microscopy pictures of Hoechst-stained nuclei and caspase 3/7-positive cells at day 13 in MSC undifferentiated or differentiated into adipocytes, in presence of 0, 1, 10, or 20 µM of phloretin. (**B**) Quantification of caspase 3/7-positive cells, by image analyses, in undifferentiated and adipocyte-differentiated MSC. Hoechst staining was used to normalize the number of caspase 3/7-positive cells in each image. * *p* < 0.05 vs. cultures no treated (0 µM of phloretin).

In addition to the expression of these two genes, the presence of caspase 3-positive apoptotic cells was studied in both undifferentiated cultures and those induced to differentiate into adipocytes at day 13, in presence of different concentrations of phloretin. In undifferentiated MSC cultures, no significant changes in the number of caspase 3-positive cells were observed with the phloretin treatments. However, in adipocyte-induced cultures, with the 10 and 20 µM concentrations, apoptotic cells increased twofold and threefold, respectively (Figure 9). These results indicate a specific apoptosis-inducing effect on adipocytes, compared to undifferentiated MSC.

### 3.6. Phloretin Affects OPG Expression and Modulates the OPG/RANKL Ratio

**Figure 10:**
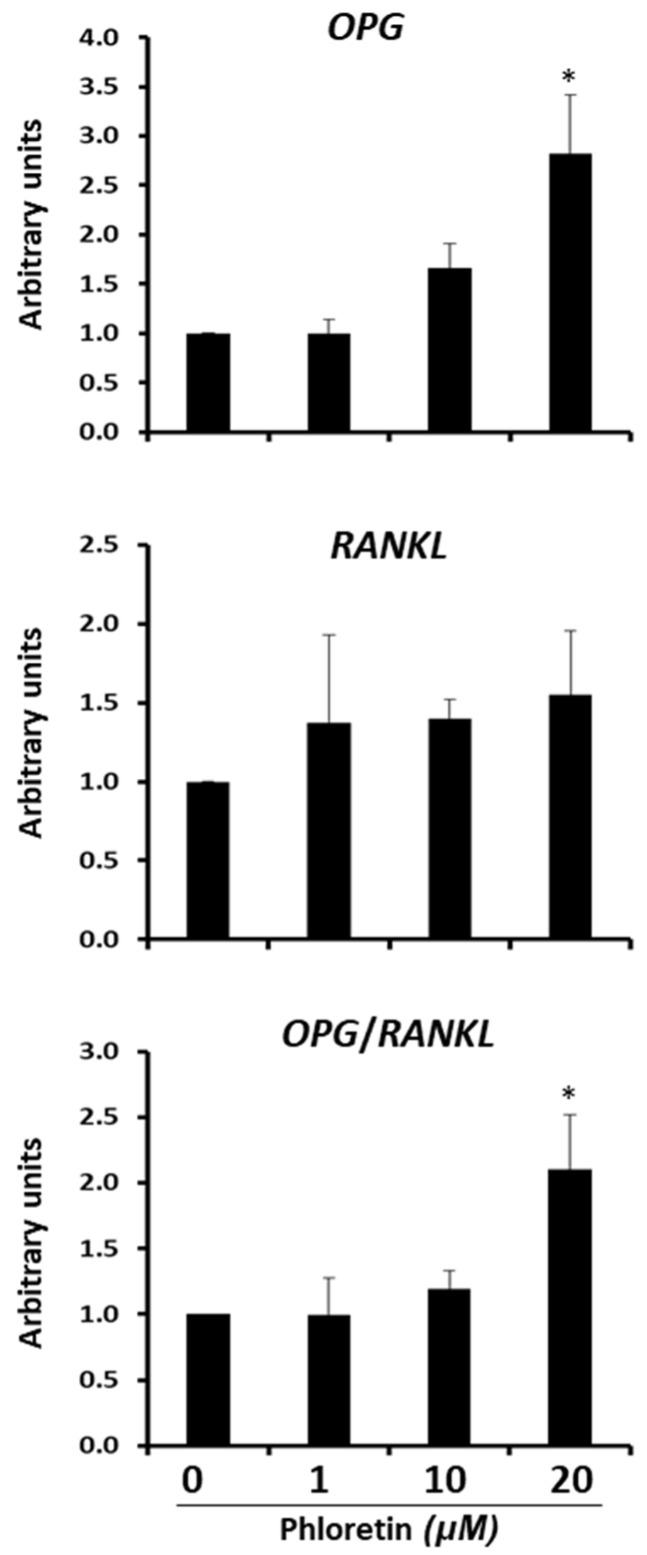
Gene expression of *OPG* and *RANKL* 13 days after beginning adipogenic differentiation of MSC cultures, in presence of 0, 1, 10, and 20 µM phloretin. *OPG/RANKL* ratio is also represented. * *p* < 0.05 vs. cultures no treated (0 µM of phloretin).

In bone marrow, adipocytes are a source of osteoprotegerin (OPG) and RANKL. OPG is a RANK decoy. It inhibits RANKL binding and activation of osteoclastogenesis. Our results showed that the differentiation of bone marrow-derived MSC into adipocytes is inhibited by phloretin. Therefore, we proceeded to study whether this flavonoid could also affect the expression of genes encoding OPG and RANKL. In MSC differentiated into adipocytes for 13 days in the presence or absence of 1, 10, or 20 µM phloretin, we observed an induction of *OPG* expression with phloretin concentration, which was significant with 20 µM (Figure 10). For *RANKL* expression, phloretin tended to increase expression, but not significantly. As a consequence, the ratio of *OPG/RANKL* expression increased significantly with 20 µM phloretin treatment (Figure 10).

## 4. Discussion

The results of this study showed that phloretin exerted an anti-adipogenic effect on human bone marrow MSC induced to differentiate into adipocytes. The formation and accumulation of lipid droplets in the cytoplasm is the main phenotypic feature displayed by adipocytes, in the late stages of differentiation [45]. Phloretin, at concentrations of 10 and 20 µM, decreased the formation of these vesicles by two to threefold. The concentrations used were in the range of those observed in plasma from rats fed with phloretin supplements (22 mg/day). In these animals, values of 54 µM were obtained 10 h after supplementation [46]. One of the properties of phloretin is its ability to inhibit glucose transporters, and thus the uptake of glucose into cells [47]. Therefore, in a first analysis of how phloretin could affect adipogenesis, two conditions of glucose concentration in the medium were evaluated: 5.5 mM (NG) and 25 mM (HG). The study of glucose uptake in cells maintained in NG medium showed a significant decrease with phloretin concentration. However, under HG conditions, no significant changes were observed. This suggests that a high glucose concentration in the medium partly counteracted the inhibitory effect of phloretin on glucose transporters. In this regard, it has been observed that increased glucose in the medium protected tumor cells from phloretin-induced apoptosis, through inhibition of type II glucose transporter [48]. Glucose availability influences adipogenic differentiation because it is required as an energy source and substrate for the formation of lipids to be stored in lipid droplets [49]. However, although HG phloretin did not significantly decrease glucose uptake, under these conditions, the inhibition of adipogenesis was similar to that obtained in the NG medium. Therefore, these data suggest other mechanisms of action of phloretin on adipogenesis, additionally to its action on glucose metabolism.

In both conditions of glucose concentration in the medium, phloretin inhibited the expression of the *PPARG2* gene in adipocytes. This gene encodes the master regulator of adipogenesis. Its upregulation at the onset of adipogenic differentiation induces expression of transcription factor C/EBPα. This factor, acting synergistically with PPARG2, activates the differentiation program toward mature adipocytes [50], regulating the expression of genes involved in lipid metabolism and in response to insulin [51]. PPARG2 activity is also critical in later stages of differentiation and in maintaining the viability of mature adipocytes. Indeed, they die in the absence of expression of such genes [52]. Therefore, its downregulation by phloretin prevents the correct process of adipogenesis and formation of lipid droplets, even when treatment is performed on mature adipocytes. In fact, expression of *LPL*, *FABP4*, and *GPD1* genes, which encode proteins involved in fat metabolism, and whose transcription is induced by PPARG, through peroxisome proliferator-activator response elements (PPARE) in their promoters [51,53,54,55], were also downregulated in cultures treated with the highest concentrations of phloretin. That was observed for both in cultures treated from the beginning of adipogenic differentiation, as well as the ones treated after 13 days of induced differentiation. This is positively correlated with the decrease in lipid-droplets formation observed in these cultures.

Another gene negatively regulated by phloretin was the one encoding GLUT4 glucose transporter. It belongs to the class I facilitative glucose transporters. Its encoding gene is expressed mainly in the heart, skeletal muscle, adipose tissue, and brain [56]. Unlike other transporters, its function is regulated by insulin. It is present in the cytoplasm of cells, in vesicles from which it is translocated into the plasma membrane, under the influence of insulin [57]. Regulation of GLUT4 transcription depends on different transcription factors, including PPARG2. Interestingly, the latter in its unliganded form represses GLUT4 transcription, by binding to its promoter. However, when PPARG2 is activated by a ligand, it separates from the promoter of the *GLUT4* gene. As a consequence, its transcription is upregulated [58]. Therefore, repression of *GLUT4* by phloretin could be mediated by the inactivation of PPARG2, although more specific studies would be needed to ascertain this possibility. Nevertheless, our results suggest that phloretin may alter glucose metabolism and insulin response in adipocytes.

Our results do not agree with some described by other authors. For instance, Hassan et al., (2007), showed that in the 3T3-L1 cell line, treatment with 50 µM phloretin induces adipogenesis, increasing triglyceride accumulation and inducing the expression of adipogenic genes, such as *PPARG* and *GLUT4* [31,59]. Nguyen et al., showed that 100 µM 3-OH phloretin inhibited adipogenesis while the same concentration phloretin-induced adipogenesis in 3T3-L1 cells [60]. Yet, such a concentration of phloretin was cytotoxic to MSC in our experiments. Recently, also in 3T3-L1 cells treated with phloretin (5 to 20 µM), an increase in adipogenesis has been described. In this work, phloretin is described as a PPARG agonist and, in contrast to what was observed in our study, as an inducer of GLUT4 expression [33]. Additionally, in pig preadipocytes, it has been described that phloretin increased adipogenesis and *GLUT4* gene expression [32]. However, in such studies, phloretin treatment of diabetic C57BL BKS-DB mouse model decreased blood glucose levels, but did not increase fat content, as might be expected based on in vitro results [32]. In the ST2 mouse preadipocyte line, an increase in lipid accumulation and adipogenic gene expression was also observed, when cells were induced to differentiate into adipocytes, in presence of 10, 50, and 100 µM of phloretin [34]. The concentrations of phloretin used by these authors are also significantly higher than those used in our study. Furthermore, in their results, it can be observed that the concentration of 10 µM had no statistically significant effect on adipogenesis. Additionally, expression of adipogenic markers at concentrations of 100 µM declined with respect to that of 50 µM. In such studies, it was observed that phloretin inhibited glucose uptake into cells differentiated into adipocytes. However, interestingly, although GLUT1 repression inhibits glucose uptake and adipogenesis, phloretin treatment of GLUT1 knocked-down cells increased adipogenic differentiation. Therefore, the authors concluded that the effect of phloretin on adipogenesis was independent of glucose uptake [34]. Yet, such data are partly contradictory to those described by other authors, which observed an increased glucose uptake in phloretin-treated adipocytes [32,33].

The discordance of research results from different authors, including the ones obtained in the present work, may be related to different experimental animal models used, and other methodologies, including culture conditions and adipogenic induction. Firstly, the cell lines used so far for the evaluation of phloretin on adipogenesis have all been preadipocytes. To the best of our knowledge, our study is the first one using MSC. Moreover, adipogenic differentiation in humans presents important differences from that in mice [61]. Thus, while clonal expansion is essential in the 3T3-L1 cell line [62], in humans this phase is not so necessary [61,63]. The differentiation protocol is also different for published works. For instance, the presence of insulin is fundamental as an inducer in the adipogenic differentiation of mouse cells. Yet, it is not required when using human MSC [64]. Thus, we did not use insulin for adipogenic induction of MSC. Such differences may influence cell responses to phloretin.

Even considering the difference between experimental models, our results confirm some observations of other authors. For example, interestingly, in the 3T3-L1 cell line, the same as that used by other authors who have described an inducing effect of phloretin on adipogenesis [31,59], concentrations of 25, 50, and 100 µM of phloretin have been described to inhibit the accumulation of lipid droplets [36]. Additionally, in 3T3-L1, extracts of Cyclopia maculata and Cyclopia subternata, which are rich in phloretin, inhibit adipogenesis [65]. In addition, phloretin treatment of 3T3-L1 cells differentiated into adipocytes decreases lipid accumulation and downregulates expression of adipogenic markers, such as PPARG [35]. These results are similar to those obtained in our study when MSC differentiated for 13 days into adipocytes were subsequently treated with phloretin.

In vivo studies have shown that phloretin prevents high-fat diets weight gain of obese mice, increases insulin sensitivity, decreases hepatic lipid accumulation, and downregulates *PPARG2* gene expression [30]. In another study also performed with obese mice induced by a high-fat diet, intraperitoneal treatment with phloretin not only prevented the weight gain of the animals but also reduced it. In the liver of these animals, phloretin inhibited lipogenesis and promoted lipolysis. Furthermore, in HepG2 cells (a human liver cancer cell line) induced with oleic acid to accumulate fat, treatment with phloretin decreased the accumulation of lipid droplets and inhibited the expression of lipogenesis-related transcription factors [37]. Similar results have also been obtained in a study with mice treated with a high-fat diet supplemented or not with phloretin. Like the previous studies, a decrease in body weight of treated mice was observed, which was accompanied by a decrease in liver, kidney, and adipose tissue weight [10]. In humans, to our knowledge, there are no such studies. Although there are numerous trials in which it has been observed that consumption of apples or juices enriched in apple polyphenols, among which phloretin stands out, is associated with a loss of body mass and visceral fat tissue [66]. Therefore, our results support these observations and suggest that inhibition of adipogenesis by phloretin may be part of the mechanism of adipose tissue loss in vivo. This suggests that the consumption of phloretin through food or as a nutraceutical may have positive effects in the treatment of pathologies such as obesity.

Cell form changes from an elongated fibroblast-like morphology into a practically spherical one, during adipogenesis. Such morphogenesis requires cytoskeleton remodeling, adapting the cytoplasm to the formation and accumulation of lipid droplets [41]. In the early stages of adipogenesis, a decrease in the synthesis of cytoskeleton components such as β- and γ-actin, as well as vimentin, α- and β-tubulin has been observed. Such changes can be greater than 90% for actin and tubulin [67,68]. This process is critical during adipogenesis. Indeed, treatment of adipose-derived stem cells in adipogenic medium with a microtubule-polymerization inhibitor, like nocodazole, promotes adipogenesis. Additionally, supplementation of adipogenic medium with taxol, which facilitates microtubule polymerization, inhibits adipogenic differentiation [68]. In our case, phloretin applied from the beginning of adipogenic induction or from day 13 to 18 after the start of differentiation increased α-tubulin synthesis in adipocytes. Although our data do not conclude whether this is a direct or indirect effect of phloretin, this fact, together with the decrease in lipid-droplet accumulation, are in agreement with the inhibitory effect of phloretin on adipogenesis.

GAPDH acts mainly in glycolysis. Because phloretin can affect glucose metabolism, GAPDH expression could be reduced in cultures treated with this flavonoid. However, our results show a tendency to increase such expression, although it is not statistically significant. This may be related to the fact that GAPDH has multiple (pleiotropic) functions in addition to its role in glycolysis. Thus, it has been described to act in cytoskeleton reorganization, as a redox sensor, in apoptosis, and in the interaction with nucleic acids, among other functions [69,70]. Therefore, there is a possibility that phloretin is inducing some of these functions in relation to GAPDH, independently of its metabolic activity.

The Wnt/β-catenin pathway plays a key role in MSC differentiation. Its activation induces differentiation into osteoblasts, while repressing PPARG2 expression. That inhibits the differentiation into adipocytes [43,71]. In adipocytes, there was an increase in beta-catenin mRNA transcription and protein translation, proportional to the concentration of the flavonoid, at day 13 of treatment with phloretin. These results can be related to those observed by other researchers. They have studied the MC3T3-E1 mouse cell line induced to differentiate into osteoblasts in the presence of the same concentrations of phloretin used in our work (1, 10, and 20 µM). Such results showed that the synthesis of β-catenin protein increased and favored osteogenic differentiation [72]. In our case, beta-catenin was induced with 10 and 20 µM phloretin at day 13, but not at day 6, after differentiation induction. This suggests that the anti-adipogenic effect of phloretin is partly mediated by the increased expression of β-catenin, mainly affecting the late stages of differentiation. In this regard, phloretin was added to differentiated cultures for 13 days, maintaining such treatment for 5 days. Results showed a significant decrease in the accumulation of lipid droplets, as well as an increase in beta-catenin. Therefore, phloretin could act mainly in stages of adipogenesis with higher metabolic activity, favoring in part lipolysis. This has been observed in experimental models using such flavonoids, like the liver of obese mice [37] and in 3T3-L1 cells differentiated into adipocytes [35]. In addition, activation of the Wnt/β-catenin pathway has also been associated with increased lipolysis [73].

Along with the increase of beta-catenin, gene expression and protein of cyclin D1 were also increased in the presence of phloretin. The gene encoding cyclin D1 is a target of beta-catenin transcriptional induction [74]. The main function recognized for cyclin D1 is cell-cycle regulation and tumorigenesis. However, cyclin D1 is involved in other cellular processes as well, such as induction of cell migration and invasion, inhibition of mitochondrial metabolism, enhancement of angiogenesis, regulation of transcription-factor signaling, and induction of chromosomal instability [75]. At the level of adipogenesis, cyclin D1 inhibits PPARG activity and decreases fat-vesicle formation. That has been observed in mouse embryonic fibroblasts differentiated into adipocytes [76,77]. Therefore, our results indicate that the effect of phloretin on adipogenesis may be partly mediated by upregulating cyclin-D1 expression, through induction of the beta-catenin pathway.

Activation of the canonical Wnt/beta-catenin pathway involves LRP5/6 co-receptors [44]. Our results show that the LRP5 coding gene was significantly induced in the presence of 20 µM phloretin at day 13. That can be positively correlated with the increase of beta-catenin. LRP6 mRNA levels tended to increase at day 6 with phloretin treatments. However, such variations were not statistically significant at any studied time of adipogenic induction. Interestingly, LRP6 is the main co-receptor of the Wnt/beta-catenin pathway in adipocytes. Besides, activation of the LRP6 gene inhibits differentiation of human MSC into adipocytes [78]. Nevertheless, under physiological conditions of adipogenic differentiation gene expression of LRP6 remains constitutive [79]. In this regard, DKK1 is an inhibitor of the Wnt/beta-catenin pathway, which acts as an antagonist by binding to LRP5/6 [44]. It is noteworthy that *DKK1* increased its expression at day 6 with the highest evaluated concentration of phloretin. Such upregulation in cultures treated with 20 µM phloretin had no effect on beta-catenin synthesis. This may be because there was also a tendency for *LRP6* expression to increase with the presence of phloretin at day 6. Thus, the ratio of *LRP6*/*DKK1* expression did not undergo significant variation. Consequently, neither did the β-catenin.

Treatment of MSC induced to differentiate into adipocytes with 10 and 20 µM phloretin resulted in a decrease in culture viability at day 13 that was not observed at day 6. Interestingly, in cells not induced to differentiate, phloretin did not affect viability at either of the two times studied. In both such differentiated and non-differentiated cells, it was observed that culture conditions in high glucose decreased cell viability, with respect to cultures grown in normal glucose conditions. That can be related to what is described by other studies, showing that bone marrow MSC cultures in high glucose concentrations increase cellular senescence [80]. This may have a negative impact on regenerative capacity under hyperglycemic conditions, mainly in diabetic patients [81].

In addition to impaired viability at day 13 in adipocyte cultures treated with phloretin, we observed downregulation of *BCL2* anti-apoptotic gene and upregulation of *BAX* apoptotic gene. Therefore, the lowered viability in these cultures was associated with increased apoptosis. Indeed, caspase activity was higher in induced cultures of adipocytes treated with the two highest concentrations of phloretin, as compared to untreated ones. However, the same concentrations of phloretin did not significantly alter the number of caspase-3 positive cells in not induced to differentiate MSC. This suggests that the effect of phloretin on cells induced to differentiate into adipocytes was specific. One possible explanation is that anabolism is activated to synthesize and accumulate lipid vesicles in the late stages of adipogenesis [82]. Thus, the presence of phloretin may interfere with the lipid and glucose metabolism of these cells, further inducing their apoptosis. Indeed, interestingly, phloretin has antitumor effects in human colorectal cancer, through downregulation of glucose-transporter 2 (GLUT2) expression. This is because tumor cells need a significant glucose supply to maintain their proliferative and metabolic capacity [3,47]. Additionally, phloretin may favor apoptosis of mature adipocytes, due to its PPARG-inhibitory capacity. As previously discussed, inhibition of this transcription factor in mature adipocytes promotes their death [52]. However, in 13-day-old adipocytes treated with phloretin for 5 days, our results showed no variation in cell-number estimation, with respect to untreated cultures. Therefore, more studies are needed to further investigate the mechanism by which phloretin induces adipocyte apoptosis. Likewise, whether this effect has a certain specificity when delivered in vivo. This could partly explain the anti-obesity effects of phloretin. In addition, it would be interesting to study whether treatment with such flavonoids in vivo could inhibit bone marrow adiposity, which is mainly associated with aging. Indeed, the use of compounds that favor adipocyte apoptosis, as we have found for the case of phloretin, has been suggested as a treatment for obesity [29].

Another interesting result of our study is that treatment with 20 µM phloretin increases the expression of the gene encoding osteoprotegerin. This produces a significant increase in *OPG*/*RANKL* gene expression ratio in such cultures. The *OPG* gene is a target of β-catenin [83]. Therefore, induction of its expression by phloretin may be mediated by β-catenin activation. Recently, it has been described that adipocytes in bone marrow can affect bone resorption, through the expression of RANKL and OPG [22,84]. Furthermore, the treatment of ovariectomized mice with phloretin protected them from bone-mass loss and increased OPG/RANKL ratio in serum [85,86]. In relation to these studies, our preliminary results suggest that the inhibitory effect of phloretin on bone resorption may be mediated, in part, by its ability to increase *OPG* expression and *OPG/RANKL* ratio in bone marrow adipocytes. Further studies are needed to confirm this possibility.

## 5. Conclusions

Our study shows for the first time that phloretin at concentrations of 10 or 20 µM decreases adipogenic differentiation of bone marrow-derived human MSC. Such adipogenic inhibition was mediated, in part, by the ability of phloretin to both increase β-catenin, as well as induce apoptosis of adipocytes in the late stages of their differentiation. Although the pharmacological use of phloretin is complicated, due to its low solubility [87], these results show that consumption of phloretin-enriched products may have positive effects for prevention and treatment of different pathologies, such as obesity. Likewise, it may have effects for decreasing bone-marrow adiposity associated with aging. Moreover, the increase of *OPG*/*RANKL* expression induced by phloretin in MSC cultures induced to differentiate into adipocytes is particularly interesting and could be related to the antiresorptive capacity of this flavonoid.

## Figures and Tables

**Table 1:** Primer sequences and product sizes.

Gene	Primer sequence (5′→ 3′)	Amplicon (bp)
Peroxisome proliferator-activated receptor gamma 2 (*PPARG2*)	GCGATTCCTTCACTGATACACTG GAGTGGGAGTGGTCTTCCATTAC	136
Lipoprotein lipase (*LPL*)	AAGAAGCAGCAAAATGTACCTGAAG CCTGATTGGTATGGGTTTCACTC	113
Fatty-acid-binding protein 4 (*FABP4*)	TCAGTGTGAATGGGGATGTGAT TCTGCACATGTACCAGGACACC	162
Glycerol-3-Phosphate Dehydrogenase 1 (*GPD1*)	ATACAGCATCCTCCAGCACAAG GGATGATTCTGCAGGCAGTG	120
Solute carrier family 2 member 4 (*GLUT4*)	CCATCCTGATGACTGTGGCTCT GCCACGATGAACCAAGGAATGG	133
Catenin Beta 1 (*CTNNB1*)	AGCTGGTGGGCTGCAGAAAATG ACAATAGCCGGCTTATTACTAGAGC	249
Cyclin D1 (*CCND1*)	CGTGGCCTCTAAGATGAAGG CCACTTGAGCTTGTTCACCA	127
LDL receptor-related protein 5 (*LRP5*)	TACTGGACAGACTGGCAGACC GTGTAGAAAGGCTCGCTTGG	209
LDL receptor-related protein 6 (*LRP6*)	TACTGGCCAAATGGACTGACT TGTTGCAAGCCAAAATGGAGT	211
Dickkopf WNT signaling pathway inhibitor 1 (*DKK1*)	ATGCGTCACGCTATGTGCT GGAATACCCATCCAAGGTGCTA	144
BCL2 apoptosis regulator (*BCL2*)	GCGCACGCTGGGAGAACAGGGT GCCCACATCTCCCGCATCCCAC	105
BCL2 associated X, apoptosis regulator (*BAX*)	TGCTCAAGGCCCTGTGCACCAAGG CGGTGGTGGGGGTGAGGAGGCT	148
TNF receptor superfamily member 11b (*OPG*)	GGCGCTACCTTGAGATAGAGTTCTG TGTTTTCTACAGGGTGCTTTAGATGAC	160
TNF superfamily member 11 (*RANKL*)	CGTCGCCCTGTTCTTCTATTTC AAATGCAGTGAGTGCCATCTTC	74
Polymerase (RNA; DNA directed) II polypeptide A (*POLR2A*)	TTTTGGTGACGACTTGAACTGC CCATCTTGTCCACCACCTCTTC	125

## Data Availability

Not applicable.
